# X-ray Photoelectron Spectroscopy Analysis of Chitosan–Graphene Oxide-Based Composite Thin Films for Potential Optical Sensing Applications

**DOI:** 10.3390/polym13030478

**Published:** 2021-02-02

**Authors:** Wan Mohd Ebtisyam Mustaqim Mohd Daniyal, Yap Wing Fen, Silvan Saleviter, Narong Chanlek, Hideki Nakajima, Jaafar Abdullah, Nor Azah Yusof

**Affiliations:** 1 Institute of Advanced Technology, Universiti Putra Malaysia, UPM Serdang, Selangor 43400, Malaysia; wanmdsyam@gmail.com (W.M.E.M.M.D.); silvansaleviter94@gmail.com (S.S.); 2 Department of Physics, Faculty of Science, Universiti Putra Malaysia, UPM Serdang, Selangor 43400, Malaysia; 3 Synchrotron Light Research Institute, Maung, Nakhon Ratchasima 30000, Thailand; n.chanlek@gmail.com (N.C.); hideki@slri.or.th (H.N.); 4 Department of Chemistry, Faculty of Science, Universiti Putra Malaysia, UPM Serdang, Selangor 43400, Malaysia; jafar@upm.edu.my (J.A.); azahy@upm.edu.my (N.A.Y.)

**Keywords:** X-ray photoelectron spectroscopy, surface plasmon resonance, thin film, quantum dot, 4-(2-pyridylazo)resorcinol, chitosan, graphene oxide

## Abstract

In this study, X-ray photoelectron spectroscopy (XPS) was used to study chitosan–graphene oxide (chitosan–GO) incorporated with 4-(2-pyridylazo)resorcinol (PAR) and cadmium sulfide quantum dot (CdS QD) composite thin films for the potential optical sensing of cobalt ions (Co^2+^). From the XPS results, it was confirmed that carbon, oxygen, and nitrogen elements existed on the PAR–chitosan–GO thin film, while for CdS QD–chitosan–GO, the existence of carbon, oxygen, cadmium, nitrogen, and sulfur were confirmed. Further deconvolution of each element using the Gaussian–Lorentzian curve fitting program revealed the sub-peak component of each element and hence the corresponding functional group was identified. Next, investigation using surface plasmon resonance (SPR) optical sensor proved that both chitosan–GO-based thin films were able to detect Co^2+^ as low as 0.01 ppm for both composite thin films, while the PAR had the higher binding affinity. The interaction of the Co^2+^ with the thin films was characterized again using XPS to confirm the functional group involved during the reaction. The XPS results proved that primary amino in the PAR–chitosan–GO thin film contributed more important role for the reaction with Co^2+^, as in agreement with the SPR results.

## 1. Introduction

Chitosan is a semicrystalline polymer material that is derived from chitin by deacetylation under alkaline condition. Chitosan is said to be the most important chitin derivative, much easier to process, and has a good mechanical and optical properties. Owing to its excellent advantages that include non-toxic, biodegradable, and biocompatible properties, as well as possessing excellent film-forming ability, chitosan has attracted many researchers to be used in various research field. For instance, chitosan has been applied in the biomedical applications including tissue engineering and drug delivery [1,2,3]. However, the stability of chitosan is low because of their hydrophilic character and also pH sensitivity [4]. Therefore, a number of techniques have been used to improve the mechanical and chemical properties of chitosan. One of the technique is by crosslinking with some reagent such as ionophore, glutaraldehyde or formaldehyde [5]. Other than that, chitosan can be reinforced by blending it with other novel material such as graphene-based substances. Graphene is the most interesting allotrope of carbon, given by its one-atom-thick layer of sp^2^-bonded carbons atom arranged into a 2D honeycomb lattice. Due to its unique 2D crystal structure, graphene can be controlled to have unlimited dimension, such as carbon materials with 0, 1, 2 and 3 dimensions [6]. In addition, there are lots of studies that have proven graphene to have remarkable strength, excellent electrical and thermal conductivity, large surface area, and biocompatibility [7,8]. Graphene can be chemically derived into graphene oxide (GO). These graphene derivatives can be easily obtained from inexpensive graphite, therefore are cost effective and also highly hydrophilic (thus, are stable in aqueous solution) which makes it easy to facilitate for the assembly of macroscopic structure [9]. Moreover, GO is known to have potential binding sites that drove to its reputation for the past decades [10,11,12,13,14,15,16,17,18,19,20,21]. Owing to the metal ion adsorption properties of both chitosan and GO, chitosan–GO based materials have received continuous attention from the scientific community. A significant amount of research has been conducted for the development of this material to match with the intended application [22,23,24,25,26,27,28,29]. In this research, 4-(2-pyridilazo)resorcinol (PAR) and cadmium sulfide quantum dots (CdS QDs) were incorporated with chitosan–graphene oxide composite material for the potential detection of cobalt ions (Co^2+^).

The compound 4-(2-pyridylazo)resorcinol is a well-known chromogenic reagent, a type of reagent that gives or changes color in a reaction [30]. This compound is a popular reagent applied in spectrophotometric, chelatometric, and colorimetric analysis due to its ability to correlate with many different metals [31]. The versatility of this compound may be contributed by its several reactive sites, such as a pyridyl nitrogen atom, azo group, and o-hydroxyl group [32]. Pyridyl is a group derived from pyridine (C_5_H_5_N) by removal of a hydrogen atom from a ring carbon atom. The removal of the hydrogen atom opens room for a bonding with the azo group (–N=N–) at the second carbon. Resorcinol (C_6_H_6_O_2_), on the other hand, is an organic compound synthesized from the destructive distillation of a natural resin. In its compound form, it appears as a white crystalline compound with a weak odor and a bittersweet taste. The reaction of resorcinol with 2-pyridylazo led to the synthesis of PAR for the first time in 1959 [33]. Since then, PAR has been widely used as a chromogenic reagent for the detection of mainly metal ions. Besides PAR, other materials such as quantum dots (QDs) are also reported to have excellent properties for potential metal ion sensing [34,35,36,37]. QDs are made up from atoms of groups II–VI, III–V, or IV–VI elements in the periodic table. In the past few decades, QDs have attracted considerable attention due to their special properties, and CdS QDs are one of the most studied QDs which are composed of semiconductors of atoms from groups II–VI [38]. There is much research information which can be searched and obtained from various sources regarding the preparation, properties, and applications of QDs [39,40,41,42,43,44]. Cadmium sulfide-based QDs (CdS QDs) are promising materials for optics, optoelectronics, medicine, and sensor technology development [45,46,47,48]. Owing to the excellent properties of these materials, both PAR and CdS QDs have been explored for their incorporation with chitosan–graphene oxide (chitosan–GO) for a potential sensing layer of cobalt ions (Co^2+^) using surface plasmon resonance (SPR) optical sensor.

Cobalt (Co^2+^) is a metal ion which is essential to human biological systems. It is one of the important components of vitamin B_12_, and is also needed as a coenzyme for cell mitosis [49]. It is present in amounts of around 1–2 mg in the human body, and can be found in the kidney, liver, heart, pancreas, and brain [50,51]. Excess intake of Co^2+^ can lead to various health effects such as dizziness, headaches, increased heart rate, asthma, and fibrosis in the lungs [52]. Therefore, it is crucial to detect Co^2+^ for continuous monitoring of this metal ion at trace amounts. Several sensors have been developed for sensing Co^2+^, which include X-ray fluorescence spectrometry (XRF), anodic stripping voltammetry (ASV), and atomic absorption spectroscopy (AAS). However, all these sensors have limitations, such as long measuring periods, expensive, and complicated sample treatments. This can be overcome using SPR sensors as an alternative, owing to its advantages such as fast measurements, simple sample preparation, cost-effectiveness, and no reference solution is necessary. 

SPR is a well-known optical sensor, which works by measuring the changes of refractive index near the thin metal film surface [53]. Any changes of the optical properties on the thin film surface will change the resonance angle. Hence, SPR can be used to detect any binding interaction, and as a sensor [54]. Interaction between molecules, such as metal ions with active layer thin films for sensing applications, have been studied extensively in the past few decades [55,56,57,58,59,60,61,62,63,64,65,66,67,68,69,70,71,72,73,74,75,76,77,78,79,80,81,82,83,84,85,86,87,88,89,90,91]. However, most of the studies only focused on the metal ion adsorption uptake performance [92]. There are limited studies on the chemical interaction and adsorption mechanism between the thin film active layer and metal ion, that become one of the main motivations in this research. X-ray photoelectron spectroscopy (XPS) is a sensitive surface analysis technique employed to explore the chemical composition on the surface of a sample. To the best of our knowledge, there was still a lack of studies on the chemical analysis of the PAR–chitosan–GO and CdS QD–chitosan–GO thin film using XPS. Therefore, in this research, XPS was used to study the surface chemistry and the interaction between PAR–chitosan–GO and CdS QD–chitosan–GO thin film with Co^2+^ as evidence for SPR sensing.

## 2. Materials and Methods

### 2.1. Prepartion of Chemicals

Medium molecular weight (MMW) chitosan with an MW of 190,000–310,000, degree of deacetylation of 75–85%, acetic acid (assay ≥ 99.7%), and 4-(2-pyridylazo)resorcinol were purchased from Sigma Aldrich (St. Louis, MO, USA); cadmium chloride decahydrate (CdCl_2_·10H_2_O), mercaptoacetic acid (MPA) (HS–CH_2_–COOH) and disodium sulfide nonahydrate (Na_2_S·9H_2_O) were purchased from R&M chemicals. The graphene oxide (GO) (4 mg/mL) was purchased from Graphenea (Cambridge, MA, USA). 

PAR (4-(2-pyridylazo)resorcinol) with a concentration of 1.5 mg/mL was prepared by diluting 0.15 g of powdered PAR with 100 mL of deionized water. To prepare the PAR–chitosan–GO composite solution, 5 mL of 1.5 mg/mL PAR was added into the mixture of chitosan–GO solution. Then, in room temperature, the mixture was stirred constantly for 24 h or until the solution mixed completely.

On the other hand, CdS QDs were synthesized by a wet method. The process began by dissolving 0.5 mmol of MPA and 0.5 mmol of CdCl_2_·10H_2_O by adding 250 mL of sterile ultra-pure water (ddH_2_O) in a 500 mL beaker. Then, the pH of the solution was adjusted to 6.0 by adding NaOH solution (1M) dropwise with constant stirring. Subsequently, the solution was purged with nitrogen gas for at least 60 min under vigorous stirring to remove excess oxygen in the solution. Sodium sulfide (Na_2_S·9H_2_O) (0.5 mmol) was then added dropwise into the stirred solution until the clear yellowish suspension of CdS QDs was obtained. The obtained aqueous CdS QDs were then quenched at 0 °C in the freezer (45 min) and stored in a refrigerator at 4 °C. CdS QD–chitosan–GO composite solution was prepared by mixing the synthesized CdS QDs with chitosan–GO solution with a 1:1:1 volume ratio.

### 2.2. Prepartion of Thin Films

Firstly, the glass slip was cleaned by using acetone or water to remove any fingerprint marks or dirt on the surface of the glass. Before the composite solution was coated onto the glass slip, the glass slip was first deposited with gold layer. The glass cover slip was deposited with a gold layer by using an SC7640 Sputter Coater (Quorum Technologies, West Sussex, UK) with the duration of 67 s for the sputtering process to obtain a 50 nm thick gold layer, which is the optimum thickness for an SPR sensor [93].

PAR–chitosan–GO and CdS QD–chitosan–GO active layers were produced by spin coating method using a P-6708D spin Coater (Inc. Medical Devices, Indianapolis, USA). Approximately 0.55 mL of the composite solution were placed on the gold layered glass film, which was spun at 6000 rpm for 30 s.

### 2.3. X-ray Photoelectron Spectroscopy

The XPS analysis was separated in two sections. The first section was carried out to investigate the existing functional groups of PAR–chitosan–GO and CdS QD–chitosan–GO thin films. The second part was to investigate the chemical changes of the PAR–chitosan–GO and CdS QD–chitosan–GO thin films after it came into contact with Co^2+^. The XPS analysis was performed in the SUT-NANOTEC-SLRI joint research facility located at the beamline in the Synchrotron Light Research Institute (SLRI), Nakhon-Ratchasima, Thailand. The samples were mounted on the stainless plate sample holder before being transferred into the vacuum chamber where the base pressure was controlled at 4 × 10^−9^ mbar. A scanning XPS Microprobe (PHI5000 Versa Probe II, ULVAC-PHI) equipped at the beamline generated X-rays from an aluminium Kα radiation at 1486.6 eV, monochromatized and focused on the sample surface at a 100 μm size spot by the quartz crystal. The energy of electrons emitted from the sample was analyzed by the concentric hemi-spherical analyzer at 45° from surface normal. The total energy resolution was typically about 0.85 eV. The charging effect was neutralized by the low-energy electrons on the sample surface. The binding energy was calibrated at the C–C peak of C 1s at 284.8 eV. The fitting procedure, was performed after the subtraction of the Shirley’s background method. The fitting was optimized in the least-square method under the conditions of constraints such as the full-width half-maximum and peak position from the reference literature [94,95]. The XPS data were fitted using Fityk fitting and data analysis software (version 0.9.8, Marcin Wojdyr, Warsaw, Poland) by Gaussian–Lorentzian functions [96].

### 2.4. Surface Plasmon Resonance Spectroscopy

SPR is an optical sensor that measured the changes of the optical properties of the thin film. Any binding interaction that occurred on the thin film surface changed the optical properties; thus, altering the resonance angle. Hence, SPR can be used to monitor any binding event optically, and also for sensing applications. SPR was previously reported to have numerous advantages, such as fast measurement, no necessity for a reference solution, cost-effective, and simple sample preparation [97]. However, SPR has low sensitivity in sensing metal ions below 100 ppm. Therefore, PAR–chitosan–GO and CdS QD–chitosan–GO have been introduced on top of the gold layer as an attempt to increase the SPR sensitivity.

**Figure 1:**
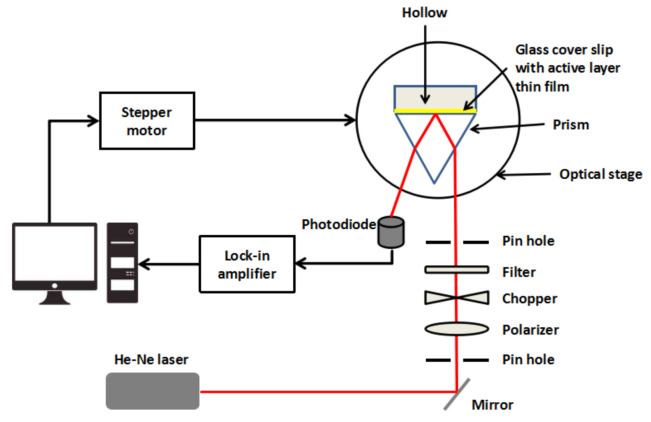
Optical setup of surface plasmon resonance spectroscopy.

To test the ability of the thin film in sensing Co^2+^, the SPR experiment setup was designed in the laboratory. The thin film was placed between the dielectric medium and the prism, as shown in Figure 1. Then, the Co^2+^ solutions of 0.01, 0.1, 1, 5, 10, 20, 40, 60, 80, and 100 ppm concentrations were inserted into the dielectric medium one at a time before the reflected beam was recorded [98]. The resonance angle was determined from the lowest peak of SPR curves for each concentration.

## 3. Results and Discussion

### 3.1. XPS Characterization of Thin Films

XPS analysis was conducted for the PAR–chitosan–GO, and CdS QD–chitosan–GO thin films in order to study the functional group that exist on the surface of each thin films. After the XPS analysis, the existence of C, O, and N elements on the PAR–chitosan–GO thin film was revealed and the ratio between elements was obtained by evaluation of the peak area. The peak area of each elements was first normalized with the corrected relative sensitivity factor (RSF) value (which includes the mean free path and the transmission function of the system) before the ratio was calculated. The ratio of C, O, and N that existed on the surface of the PAR–chitosan–GO thin film was 66.41%, 27.96%, and 5.63%, respectively, as shown in Table 1. The deconvolution of the peak for each elements that existed on the surface of the thin films was also carried out to investigate the sub-peak component using the Gaussian–Lorentzian function based on theoretical prediction and previously reported works from the High Resolution XPS of Organic Polymers: The Scienta ESCA300 Database [99].

**Figure 2:**
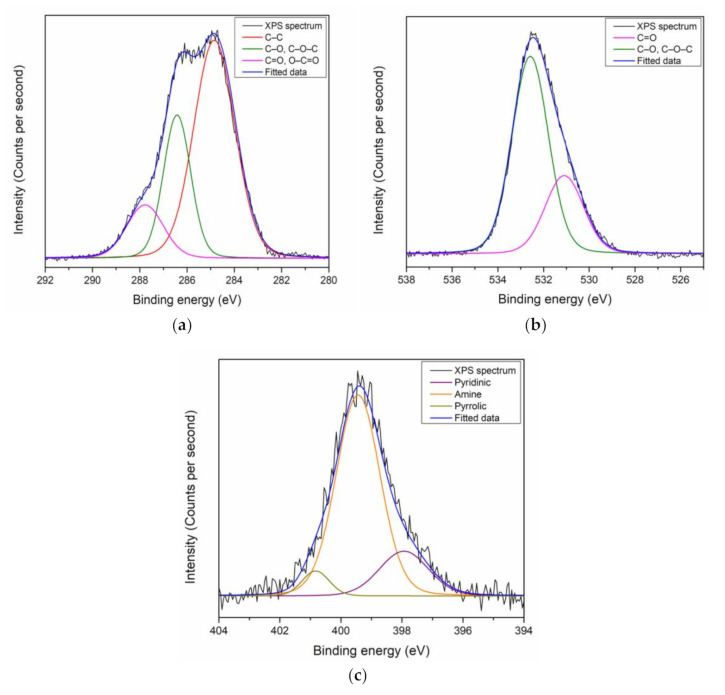
XPS narrow scan spectra of PAR–chitosan–GO for (**a**) C 1s; (**b**) O 1s; and (**c**) N 1s.

The C 1s peak for PAR–chitosan–GO thin film was mainly resolved into C–C bonds and C–O or C–O–C bonds at 284.7 eV and 286.6 eV, respectively, as shown in Figure 2a. A lower intensity of C=O or O–C=O bonds was also deconvoluted at 288.1 eV. The existence of C–O or C–O–C and C=O or O–C=O from the deconvolution of the C 1s peak indicated the existence of the oxygen-containing group in GO’s structure, and has a good agreement with the theoretical structure of GO [100]. Moreover, the deconvolution of the O 1s peak by Gaussian–Lorentzian function was carried out to confirm the deconvolution of oxygen-containing functional groups of the C 1s peak, as shown in Figure 2b. The O 1s spectra for the gold/GO thin film were deconvoluted into two sub-peaks at 530.9 eV and 532.9 eV that were assigned to C=O and C–O or C–O–C bonds, respectively. For the N 1s peak, the peak was deconvoluted into three sub-peaks at 397.9 eV, 399.7 eV, and 400.9 eV, which were assigned for pyridinic, amine, and pyrrolic N, respectively, as shown in Figure 2c. The deconvoluted N 1s peak had a good agreement with the N functional groups that exist on PAR and chitosan [101].

**Figure 3:**
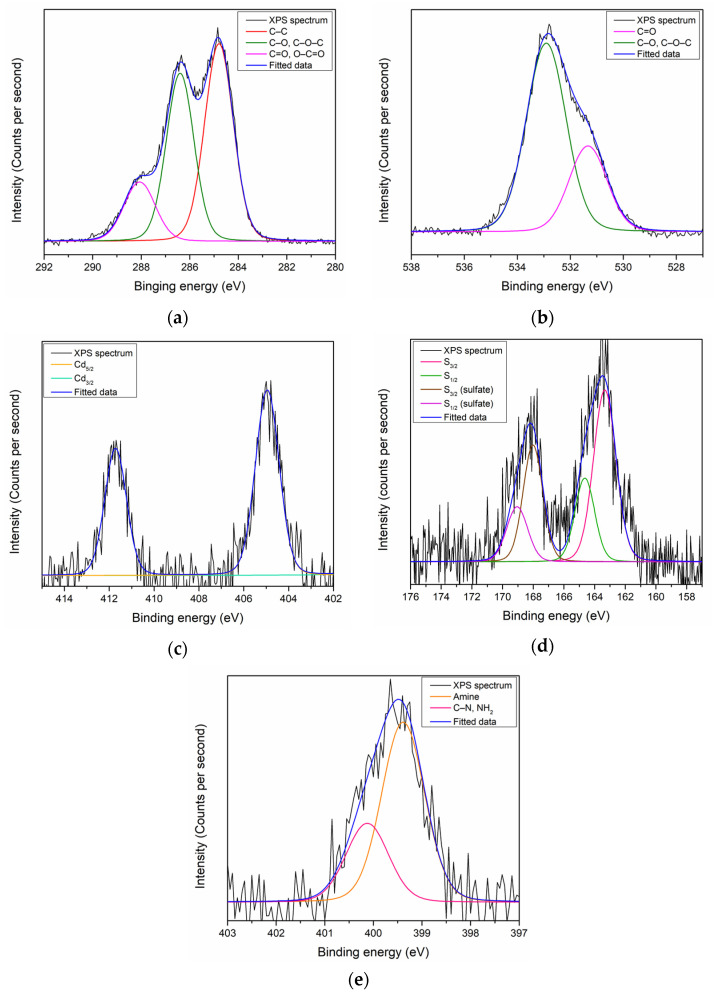
XPS narrow scan spectra of CdS QD–chitosan–GO for (**a**) C 1s; (**b**) O 1s; (**c**) Cd; (**d**) S 2p; and (**e**) N 1s.

On the other hand, the XPS analysis revealed the existence of C, O, Cd, S, and N on the surface of the CdS QD–chitosan–GO thin film with ratios of 67.53%, 28.63%, 0.24%, 1.65%, and 1.95%, respectively [102]. The quantitative result is summarized in Table 2. Each elements peak that existed on the surface of the CdS QD–chitosan–GO thin film was also deconvoluted using the Gaussian–Lorentzian function to obtain the information on the sub-peak component. The C 1s peak for the CdS QD–chitosan–GO thin film can be resolved into three sub-peak components. The first and second peaks at 284.6 eV and 286.8 eV were assigned to a C–C bond and C–O or C–O–C bonds, respectively, while the third peak at 288.1 eV could be assigned to C=O or O–C=O bonds, as shown in Figure 3a. For the O 1s, the peak at 531.1 eV and 533.1 eV could be resolved into C=O and C–O or C–O–C, as shown in Figure 3b. Moreover, the Cd 3d peak could be deconvoluted into two components. The first component at 405 eV was assigned to Cd_5/2_, while the second peak at 412 eV could be assigned to Cd_3/2_, as shown in Figure 3c. For the deconvolution of the S 2p peak, two pairs of sub-peaks were revealed, as shown in Figure 3d. The first pair at 163.3 eV and 164.6 eV, assigned to S_3/2_ and S_1/2_, respectively, corresponds to the S–Cd bond [103]. The second pair also contained S_3/2_ and S_1/2_ sub-peaks: 167.8 eV and 169.1 eV, respectively. The existence of the second pair was related to the presence of residual sulfate on the surface of the CdS QD–chitosan–GO thin film that might not have completely been removed during preparation [104]. The existence of Cd and S elements on the surface of thin film proved that the combination of CdS QDs with the chitosan–GO was successful. For the last element, the N 1s could be resolved into two sub-peaks, which were the amine groups and C–N or NH_2_ bonds at 399.2 eV and 400.2 eV, respectively, as shown in Figure 3e [92].

### 3.2. SPR Response of Thin Films With Co^2+^

**Figure 4:**
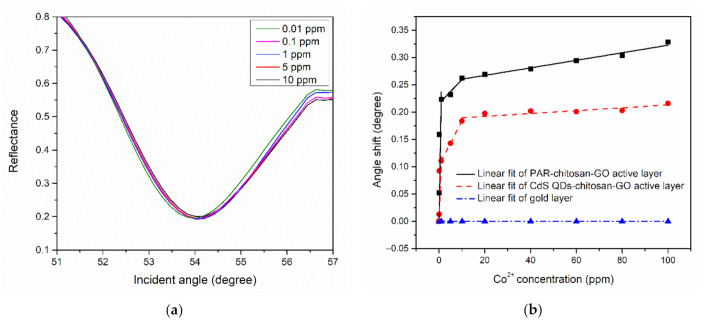
(**a**) SPR response of thin films in contact with Co^2+^ at 0.01–10 ppm concentration, and (**b**) the comparison of the linear regression analysis for various concentration of Co^2+^ in contact with gold layer and modified gold layer thin films.

The SPR analysis began by finding the SPR curve of the thin films when in contact with Co^2+^ solution at 0.01, 0.1, 1, 5, 10, 20, 40, 60, 80, and 100 ppm concentrations. The resonance angle then was determined from each of the SPR curves, as shown in Figure 4a [105]. The SPR curves for both PAR–chitosan–GO and CdS QD–chitosan–GO thin films were shifted to the right when Co^2+^ solution was used, with the lowest detection of 0.01 ppm for both thin films. This result proved that the binding event did occur between the Co^2+^ and the modified gold thin films [106]. 

The shift of the resonance angle has been applied as a parameter to measure the sensitivity of the SPR sensor [107]. The comparison of the resonance angle shift of gold, PAR–chitosan–GO, and CdS QD–chitosan–GO thin films towards various concentration of Co^2+^ is shown in Figure 4b. From the figure, it can be observed that there are no changes in the resonance angle when the gold layer is introduced with different concentrations of Co^2+^. The figure clearly demonstrates the sensitivity enhancement of the active layers compared to only the gold layer film. To be exact, the linear regression analysis revealed that the sensitivity of the active layers towards Co^2+^ are different; the PAR–chitosan–GO thin film exhibited a greater slope compared to the CdS QD–chitosan–GO thin film with slopes of 0.2370° ppm^−1^ and 0.1188° ppm^−1^, respectively, for lower concentrations (0 ppm to 1 ppm). At high concentrations (10 ppm to 100 ppm), PAR–chitosan–GO thin film exhibited a greater slope at 0.00069° ppm^−1^ compared to the CdS QD–chitosan–GO thin film with a slope of 0.00026° ppm^−1^. These results confirmed that the PAR–chitosan–GO thin film attracts more Co^2+^ compared to the CdS QD–chitosan–GO thin film [108].

**Figure 5:**
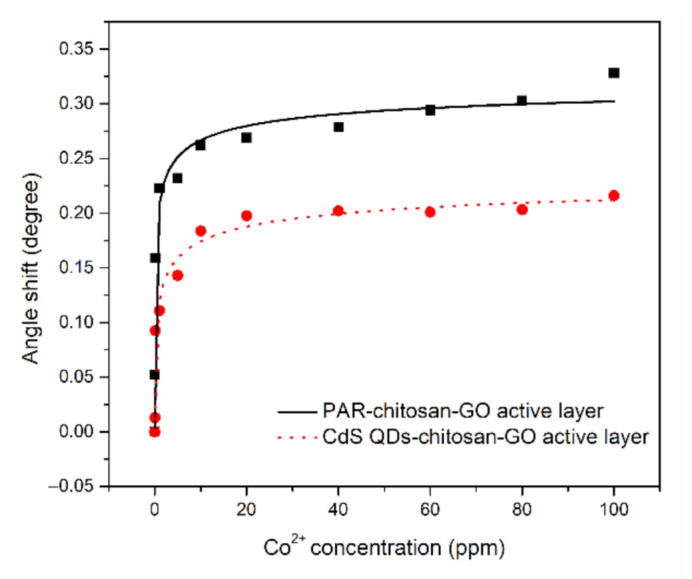
SPR angle shift comparison (fitted with Sips model) between PAR–chitosan–GO and CdS QD–chitosan–GO thin films in contact with different concentration of Co^2+^ from 0 to 100 ppm.

Furthermore, the shift of resonance angle data was fitted by Langmuir–Freundlich models to obtain the binding affinity of Co^2+^ ions with the thin films [109,110,111,112]. The Langmuir–Freundlich models can be expressed as $\Delta \theta =\frac{\Delta \theta_{\max}{\left(K_{a}C\right)}^{n}}{1+{\left(K_{a}C\right)}^{n}}$, where ∆*θ*_max_ is the maximum of the resonance angle shift, *K_a_* is the affinity constant, *C* is the concentration of the Co^2+^ ions, and *n* is the heterogeneity index. As shown in Figure 5, it can be observed that the SPR angle shift of the PAR–chitosan–GO thin film produced a greater maximum angle shift compared to the CdS QD–chitosan–GO thin film. The maximum resonance angle shifts for the PAR–chitosan–GO and CdS QD–chitosan–GO thin films were 0.334° and 0.213°, respectively. Resonance angle shift was caused by the changes in the material composition, and the higher the angle shift, the greater the changes of the material composition [113,114]. Other than that, the binding affinity constant of PAR–chitosan–GO thin films were higher as compared to the CdS QD–chitosan–GO thin films, i.e., 1.649 ppm^−1^ and 0.939 ppm^−1^, respectively, with R^2^ of 0.96. The calculated fitting parameters are summarized in Table 3. Overall, the results obtained from the binding model fitting also show a great response for the adsorption of Co^2+^ ions with the composite sensor layer.

### 3.3. Evidence of Co^2+^ Interaction with the Thin Films

After the SPR analysis, XPS analysis was carried out to study the changes of the surface chemistry on the thin films. The PAR–chitosan–GO thin film that was in contact with Co^2+^ (1 ppm) was analyzed by XPS, and the results revealed that the same elements were detected: C, O, N, and an extra peak for Co at 63.51%, 29.32%, 6.94%, and 0.22% ratio, respectively [92]. The ratio for each element is recorded in Table 4. Each element peak was then deconvoluted using the Gaussian–Lorentzian function to investigate the changes of functional groups on the thin film surface. 

**Figure 6:**
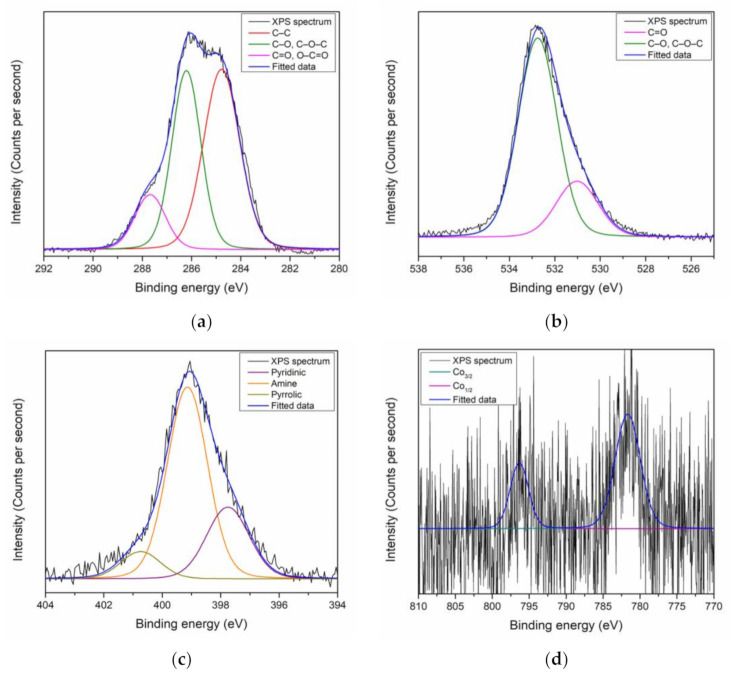
XPS narrow scan spectra of PAR–chitosan–GO after contact with Co^2+^ for (**a**) C 1s; (**b**) O 1s, (**c**) N 1s; and (**d**) Co 2p.

Identical to the C 1s before it was in contact with Co^2+^, the C 1s peak for the PAR–chitosan–GO thin film was resolved into three sub-peaks at 284.7 eV, 286.8 eV, and 287.9 eV, which were assigned to a C–C bond, C–O or C–O–C bonds, and C=O or O–C=O bonds, respectively, as shown in Figure 6a. The ratios of the chemical composition were also calculated to provide a better description on the changes observed for the XPS results. When compared with the C 1s of the PAR–chitosan–GO thin film before it was in contact with Co^2+^, there was decrease in the sub-peak intensity for the C 1s spectra after the interaction. The ratio for the sub-peak at 287.9 eV that was assigned to the O–C=O bond decreased from 13.31% to 12.61% after it was in contact with Co^2+^. Decreasing of this sub-peak indicated that the chemical state of the O–C=O functional groups undergo changes after the interaction. Moreover, the intensity for the sub-peak at 286.8 eV that was assigned to the C–O or C–O–C bond increased due to the decrease in the sub-peak intensity for the C=O or O–C=O bond. As the result, the calculated ratio for the C–C bond decreased from 60.28% to 48.68%. 

To confirm the changes of the sub-peak component, O 1s after the interaction with Co^2+^ was also fitted using the Gaussian–Lorentzian function. For the O 1s peak, the deconvolution of the spectra also comprised two sub-peaks at 530.9 eV and 532.9 eV, which were assigned to C=O and C–O or C–O–C bonds, respectively. As shown in Figure 6b, the sub-peak at 532.9 eV that was assigned to C=O decreased, which was also proved by calculating the ratio. The ratio of C=O decreased from 29.5% to 23.15%. This result is in good agreement with the changes of the sub-peak of C 1s after it was in contact with Co^2+^. The O–C=O can lose a proton and form a negatively charged ion (COO^−^). The existence of these negatively charged functional groups may attract the positively charge Co^2+^ to form electrostatic interactions; thus, changing the optical properties of the thin film surface [106]. Moreover, the XPS results also proved that Co^2+^ may have interacted electrostatically with the amino group that existed on the surface of the PAR–chitosan–GO thin film [92]. The N 1s peak was deconvoluted into three sub-peaks at 397.9 eV, 399.3 eV, and 400.8 eV, which were assigned to pyridinic, amine, and pyrrolic N, respectively, as shown in Figure 6c. From the plotted graph, there was decrease in the amine functional group that existed on the surface of the PAR–chitosan–GO thin film. The amine group decreased from 74.81% to 65.01% after contact with Co^2+^. The XPS analysis also revealed an extra peak that was assigned to Co 2p, that proved the existence of Co on the surface of the PAR–chitosan–GO thin film, as shown in Figure 6d. The Co 2p peak consisted of two sub-peaks that were assigned to Co_3/2_ at 781.7 eV, associated with the Co^2+^ oxidation state, while the second peak at 796.4 eV was assigned to Co_1/2_. The quantitative results are summarized in Table 5.

**Figure 7:**
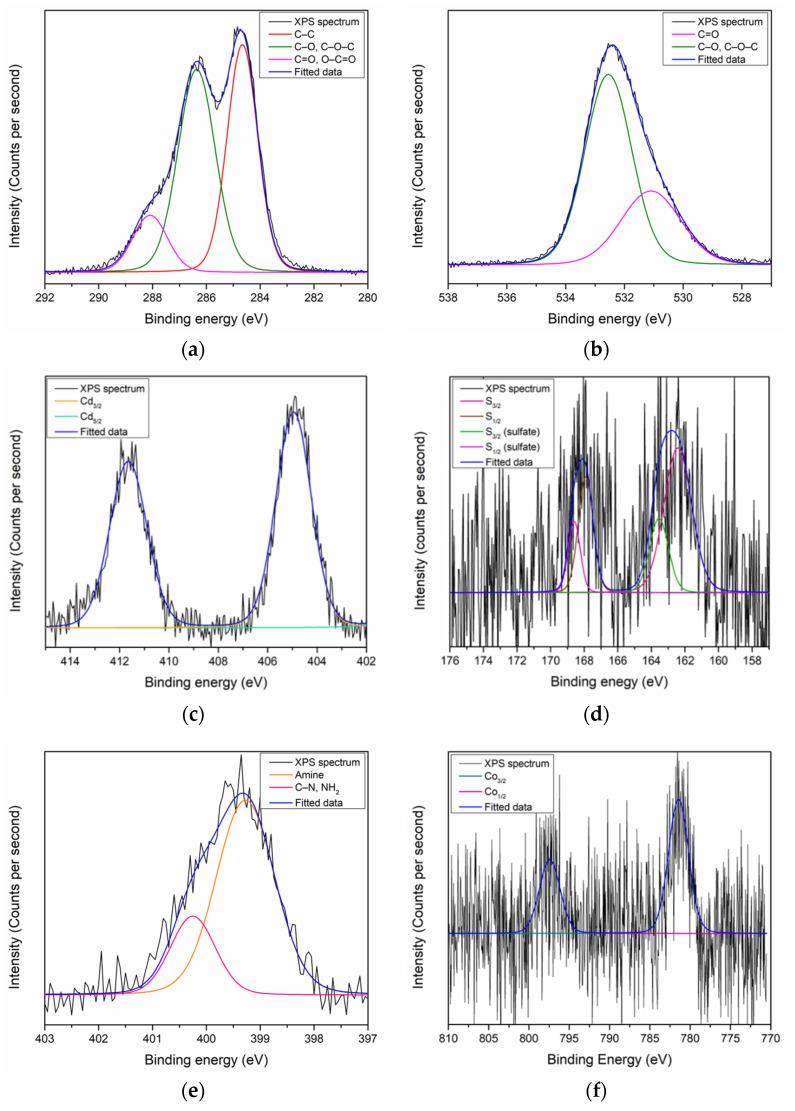
XPS narrow scan spectra of CdS QD–chitosan–GO after contact with Co^2+^ for (**a**) C 1s; (**b**) O 1s; (**c**) Cd; (**d**) N 1s; (**e**) S 2p; and (**f**) Co 2p.

Moving on to the XPS results of the CdS QD–chitosan–GO thin film after it came into contact with Co^2+^: the surface of the CdS QD–chitosan–GO thin film was identified with C, O, Cd, S, and N at 66.6%, 28.05%, 0.27%, 2.25%, and 2.59% ratios, respectively, as shown in Table 6. An extra peak of Co at a 0.29% ratio was also detected, proving the existence of Co at the surface of the thin film. The first element, C 1s, was deconvoluted into the three sub-peak components. The C 1s of the CdS QD–chitosan–GO thin film comprised C–C, C–O or C–O–C, and C=O or O–C=O bonds first at 284.7 eV, 286.8 eV, and 288.1 eV, respectively, as shown in Figure 7a. Interestingly, after the CdS QD–chitosan–GO thin film was in contact with Co^2+^, the sub-peak component at 288.1 eV that was assigned to C=O or O–C=O decreased. The ratio for this sub-peak decreased from 15.24% to 12.12% after it came into contact with Co^2+^. Moreover, the O 1s peak of CdS QD–chitosan–GO thin film after contact with Co^2+^ could also be resolved into two sub-peaks at 531.0 eV and 532.9 eV, which were assigned to C=O and C–O or C–O–C, respectively, as shown in Figure 7b. From the deconvolution of the O 1s peak after contact with Co^2+^, it was confirmed that the peak intensity at 532.9 eV decreased when the ratio changed from 30.43% to 26.71%. Furthermore, no changes occurred after the Cd 3d peak came into contact with Co^2+^. The Cd 3d peak could be resolved into two sub-peaks at 405 eV and 412 eV, which were assigned to Cd_5/2_ and Cd_3/2_, respectively, as shown in Figure 7c. For S 2p, two pairs of sub-peak components were identified. The first pair at 162.4 eV and 163.5 eV was assigned to S_3/2_ and S_1/2_, respectively. The second pair that corresponds to the presence of residual sulfate on the surface was also assigned to S_3/2_ and S_1/2_, at 167.8 eV and 168.5 eV, respectively, as shown in Figure 7d. For N 1s, two sub-peaks were deconvoluted at 399.2 eV and 400.2 eV, which were assigned to the amine groups and C–N or NH_2_ bonds, respectively, as shown in Figure 7e. The sub-peak ratio of N 1s that was assigned to the amine functional group also decreased from 70.01% to 67.32%. Furthermore, one spin-orbit pair corresponding to the Co^2+^ oxidation state was identified at 781. 5 eV and 797.3 eV, which was assigned to Co_3/2_ and Co_1/2_ sub-peaks, respectively, as shown in Figure 7f. 

Similarly to the PAR–chitosan–GO thin film, it is believed that Co^2+^ interacts with negatively charged functional groups on the surface of the CdS QD–chitosan–GO thin film, i.e., COO^−^ and the amine functional groups through electrostatic interaction. Moreover, it is also suggested that PAR played more important role during the interaction, based on the XPS and SPR results. The surface of the PAR–chitosan–GO thin film contained more active sites for interaction with Co^2+^ owing to the presence of PAR; it provides more amine functional groups. As a result, more Co^2+^ could be absorbed on the surface of the PAR–chitosan–GO thin film. The existence of more active sites makes PAR–chitosan–GO thin film more advantageous in sensing Co^2+^ compared to CdS QD–chitosan–GO thin film; this was proven using SPR where the PAR–chitosan–GO thin film had a higher binding affinity towards Co^2+^. The quantitative results are summarized in Table 7.

## 4. Conclusions

In this study, PAR–chitosan–GO and CdS QD–chitosan–GO thin films have been characterized using X-ray photoelectron spectroscopy (XPS), before and after interaction with Co^2+^. The XPS results revealed the existence of C, O, and N on the surface of the PAR–chitosan–GO thin film, while C, O, Cd, S, and N were detected on the surface of the CdS QD–chitosan–GO thin film. Each element peak has been deconvoluted by a Gaussian–Lorentzian curve fitting program in order to determine the sub-peak components. Further analysis of the PAR–chitosan–GO and CdS QD–chitosan–GO thin films using SPR has also been carried out; both thin films were able to detect Co^2+^ as low as 0.01 ppm. The binding affinity constant of PAR–chitosan–GO thin film was higher compared to the CdS QD–chitosan–GO thin film, i.e., 1.649 ppm^−1^ and 0.939 ppm^−1^, respectively, with R^2^ of 0.96. After interaction with Co^2+^, the thin films were characterized again by XPS to confirm the chemical interactions involved between the Co^2+^ with the existing functional groups on the thin film surfaces. From the deconvolution of each element, Co^2+^ may have interacted electrostatically with negatively charged functional groups on the surface of the both chitosan–GO thin films, i.e., COO^−^ and amine groups. Moreover, it is suggested that PAR played more important role during the interaction, based on the XPS and SPR results. The surface of the PAR–chitosan–GO thin film contained more active sites due to the presence of PAR, which provided more amine functional groups. The existence of more active sites promote the use of PAR–chitosan–GO thin film in sensing Co^2+^ compared to CdS QD–chitosan–GO thin film.

## Figures and Tables

**Table 1:** Elemental composition of the PAR–chitosan–GO thin film. RSF, relative sensitivity factor.

Sample	Element	Corrected RSF	Area	Corrected Area	Ratio (%)
PAR–chitosan–GO	C	1.2070	15,701	13,008.3	66.41
	O	3.1048	17,005	5477.0	27.96
	N	2.0520	2264	1103.3	5.63

**Table 2:** Elemental composition of the CdS QD–chitosan–GO thin film.

Sample	Element	Corrected RSF	Area	Corrected Area	Ratio (%)
CdS QD–chitosan–GO	C	1.2070	15,033	12,454.8	67.53
	O	3.1048	16,396	5280.9	28.63
	Cd	25.9923	1147	44.1	0.24
	S	2.4319	742	305.1	1.65
	N	2.0520	738	359.6	1.95

**Table 3:** The calculated fitting parameters using Langmuir–Freundlich models.

Sample	*K* (ppm^−1^)	R^2^	*n*	$\Delta \theta_{max}$ (°)
PAR–chitosan–GO	1.649	0.96	0.400	0.334
CdS QD–chitosan–GO	0.939	0.96	0.400	0.213

**Table 4:** Elements composition of the PAR–chitosan–GO thin film after it was in contact with Co^2+^.

Sample	Element	Corrected RSF	Area	Corrected Area	Ratio (%)
PAR–chitosan–GO	C	1.2070	16,139	13,371.2	63.51
	O	3.1048	19,169	6174	29.32
	N	2.0520	2998	1461	6.94
	Co	18.5764	858	46.2	0.22

**Table 5:** Chemical composition ratio for C 1s, O 1s, and N 1s for the PAR–chitosan–GO thin film before and after contact with Co^2+^.

Sample	Element	Corrected RSF	Sub-Peak	Area	Corrected Area	Ratio (%)
PAR–chitosan–GO before contact with Co^2+^	C	1.2070	C–C	9491.2	7863.5	60.28
			C–O, C–O–C	4159.1	3445.8	26.41
			C=O, O–C=O	2095.1	1735.8	13.31
	O	3.1048	O–C, C–O–C	11,196.8	3606.3	70.50
			O=C	4685.2	1509.0	29.50
	N	2.0520	Pyridinic	442.4	214.6	19.41
			Amine	1705.1	830.9	74.79
			Pyrrolic	132.3	64.5	5.80
PAR–chitosan–GO after contact with Co^2+^	C	1.2070	C–C	7526.9	6236.0	48.68
			C–O, C–O–C	5985.3	4958.8	38.71
			C=O, O–C=O	1949.8	1615.4	12.61
	O	3.1048	O–C, C–O–C	14,541.3	4683.5	76.80
			O=C	4392.7	1414.8	23.15
	N	2.0520	Pyridinic	772	376.21	25.87
			Amine	1939.9	946.29	65.01
			Pyrrolic	272.1	132.60	9.12

**Table 6:** Elemental composition of the CdS QD–chitosan–GO thin film after contact with Co^2+^.

Sample	Element	Corrected RSF	Area	Corrected Area	Ratio (%)
CdS QD–chitosan–GO	C	1.2070	18,606	15,415.1	66.60
	O	3.1048	20,161	6493.5	28.05
	Cd	25.9923	1608	61.9	0.27
	S	2.4319	1267	521	2.25
	N	2.0520	1230	599.4	2.59
	Co	18.5764	1070	57.6	0.29

**Table 7:** Chemical composition ratios for C 1s, O 1s, and N 1s for the CdS QD–chitosan–GO thin film before and after contact with Co^2+^.

Sample	Element	Corrected RSF	Sub-Peak	Area	Corrected Area	Ratio (%)
CdS QD–chitosan–GO before contact with Co^2+^	C	1.2070	C–C	6594.5	5463.5	46.45
			C–O, C–O–C	5438.9	4506.1	38.31
			C=O, O–C=O	2163.6	1792.5	15.24
	O	3.1048	O–C, C–O–C	10,782.7	3472.9	69.57
			O=C	4716.3	1519.0	30.43
	N	2.0520	Amine	515.9	251.4	70.01
			C–N	221.1	107.7	29.91
CdS QD–chitosan–GO after contact with Co^2+^	C	1.2070	C–C	7542.7	6249.1	44.56
			C–O, C–O–C	7332.8	6075.2	43.32
			C=O, O–C=O	2051.6	1699.8	12.12
	O	3.1048	O–C, C–O–C	14,528.3	4679.3	73.29
			O=C	5294.7	1705.3	26.71
	N	2.0520	Amine	935.1	455.7	67.32
			C–N	453.9	221.2	32.68

## Data Availability

Not applicable.
